# Effect of High Temperatures on the Impact Strength of Concrete Based on Recycled Aggregate Made of Heat-Resistant Cullet

**DOI:** 10.3390/ma13020465

**Published:** 2020-01-18

**Authors:** Aleksandra Powęzka, Jacek Szulej, Paweł Ogrodnik

**Affiliations:** 1 Faculty of Security Engineering and Civil Protection, Main School of Fire Service, 01-629 Warsaw, Poland; 2 Faculty of Civil Engineering and Architecture, Lublin University of Technology, 20-618 Lublin, Poland; j.szulej@pollub.pl; 3 Institute of Security Engineering, Main School of Fire Service, 01-629 Warsaw, Poland; pogrodnik@sgsp.edu.pl

**Keywords:** concrete, recycled aggregate, heat resistant cullet, concrete impact strength

## Abstract

The article presents results obtained during testing of concrete based on CEM I 42.5R Portland cement, fine and coarse aggregate, glass, volatile ash, and superplastifier. The concrete mixture was modified using filler consisting of bromosilicate heat resistant cullet. Recycled aggregate was added to the batch. Samples for the need of testing were produced as (100 × 100 × 100) mm cubes. Before commencing proper tests, samples have been heated within the temperature range of 20–800 °C. Tests carried out during the proper testing procedure included tests of compressive strength, elevated temperature, impact strength, as well as macroscopic tests of the contact area. The obtained test results have provided proof of there being a possibility of producing special concrete, modified by products obtained from heat resistant cullet. This type of is generally characterized by satisfactory performance parameters. The average compressive strength for concrete modified by a 10% of heat resistant cullet was determined as 43.6 MPa and 48.3 MPa respectively after 28 and 180 days of curing.

## 1. Introduction

A particularly challenging issue facing different countries worldwide is to find ways to stop the degrading impact of the population on the surroundings. Two phenomena that arise from uncontrolled economic and civilizational growth and development are exerting a hazardous impact on the natural environment. The first disquieting phenomenon is the generation of all types of waste, and the second one is the depletion of natural resources. Both phenomena require global attention to allow innovative and sustainable applications being worked out [1].

Mass-scale consumptionism goes back to the 19th and 20th centuries. It has caused an uncontrolled economic growth worldwide and wasting of natural resources [2,3,4].

The latest Eurostat results from May 2017 determine the percentage of particular economic sectors in total generation of waste in the European Union (EU). The building sector generated 34.7% of the total amount of waste in 2014. At that time, the EU generated 2503 million tonnes of waste. The subsequent positions were occupied by mining and quarrying (28.2%), production (10.2%), services related to water supply and sewage disposal management (9.1%), and households (8.3%). The remaining 9.5% comprised waste generated by other types of economic activity, primarily service (3.9%) and power sectors (3.7%) [5].

The availability of natural raw materials is of a great social importance. The strategy of rational resource management is an interesting research issue based on the sustainable development principle. Recycling and re-use of natural raw materials are the main focus here. Researchers show considerable interest in possibilities of using recovered aggregate. The absence of clear guidance in this respect limits the usage of those materials and causes their marginalisation. According to standard [6] aggregate obtained from recycling is fully valuable material, which requires testing and assessment. It is also a good method for improving both ecological and economic aspects of the concrete production sector [7,8,9,10].

In its support of sustainable development in the construction sector, in 2015 the European Commission adopted a plan relating to a closed loop economy. The innovative concept consisted in introducing a closed service life of a product. The product is not thrown out, and as an effect to the landfill after the end of its service life it is subject to recovery and recycling. Stockpiled waste may to a large extent be used as secondary raw materials. Processing of waste can reduce the generated amounts [11,12,13,14,15].

Numerous research studies have provided proof of the suitability of using aggregate obtained from recovery. According to estimations of the Polish Association of Aggregate Producers the value of this production is ca. 4.5 million tonnes, which comes up to 2.5% of all aggregate amount used in Poland [16]. The use of crushed waste materials, such as for example glass, ceramics, or concrete obtained from demolition works as aggregate for concrete composites can help minimize their adverse impact on the environment. The importance of recycled aggregate keeps growing, because it changes the properties of concrete. Before their use in a concrete mixture, recycled materials need to undergo testing [1,13,17].

In Denmark, designing of ecological concrete was initiated already in 1998, thanks to which the waste from other processes may be used as a raw material. This can limit the volume of production waste and reduces the obtaining of aggregate from natural deposits [18,19,20].

The process of obtaining recycled aggregate from concrete is complicated, and it comprises assessing concrete source, selective facility demolishing, crushing larger elements, removal of any present contamination, initial and elementary concrete crushing, fractionation, and the improvement process [21].

Cullet waste stored in piles does not decompose [22,23]. It is, however, a perfect recycled raw material that is resistant to mould and humidity. The construction industry generated many possibilities for the recycling of glass. Crushed glass may be considered an alternative solution for natural aggregate. Already years ago, certain countries have introduced innovative building products which contained cullet first subjected to processing [24,25,26]. Cullet added to a concrete mixture keeps causing diverse problems to process engineers and producers, as it has an effect on diverse properties, both physical and mechanical ones—for example it extends the initial and final setting time, and delays hydration. For years now researchers have been studying the impact of the amount of added crushed recycled aggregate on concrete strength. Results compiled and presented in studies [14,21,27,28,29,30,31,32] do not provide an unequivocal assessment of the impact exerted by cullet on concrete strength. Researchers argue that as the contents of recyclate are increased, the compressive strength of concrete tends to drop. The strength grows quicker than in the case of concrete based on natural aggregate. The quality of obtained composite is affected by the moisture content of recyclate [33], implementation method, contents of contamination, and raw material used to produce the recyclate. It is recommended to have the coarse fraction of recyclate limited to 30% to help guarantee better workability.

Numerous studies [34,35,36,37,38,39,40,41] attempt to find ways of using materials for the production of ceramics, binders, mortar, concrete, insulation materials, for the production of glass fibers, foamed glass, mats, insulation plates, laminates and bituminous materials. Tests [10,40,42] were carried out to determine possibilities of applying cullet among others as replacement for natural aggregate to concrete, substitute of natural soils, or construction of roads and subgrades.

For needs of the testing procedure use was made of cullet coming from diverse sources, such as for example float type window glazing, car windows, tempered glass, crystal glass, mirrors, colorless, green and brown packaging glass, and kinescope glass. The cullet may be used as a substitute of cement, sand, and coarse aggregate. Research of available literature has shown that if cement is replaced by cullet, after 28 days of curing concrete composite has a lowered compressive strength as compared to reference concrete. On the other hand, after 90 and 180 days of curing a much smaller impact of the presence of cullet is observed on lowering the strength of composites [1]. This proves the pozzolanic reactivity of cullet [35], which is correlated with the average compressive strength. To be able to use cullet as substitute of fine aggregate, it is necessary to take into consideration the type of applied glass. If 10% and 30% of cullet is added, a decrease in strength of 10% and 25% respectively takes place for the majority of samples subjected to testing. Results of conducted tests point to a decrease in strength of the composite as compared to the performed control test [31]. The produced samples had different shapes and dimensions.

Observations of the topography of studied material under a scanning electron microscope (SEM) allow the presumptions that recycled waste material that contain highly resistant glass is characterized by a denser microstructure as compared to a common sample [43]. Furthermore, attention should be drawn to the size of particles and the content of glass aggregate on concrete properties.

Concrete structures may be subjected to dynamic loads caused by such phenomena, as earthquakes, waves, wind, and machinery. This necessitates making a correct assessment of dynamic response of the structure to this type of load. Researchers have studied the major part of strength properties of concrete, however, literature still contains insufficient research concerning tests of such dynamic features, as the vibration damping factor [44].

Researchers [8,45,46,47,48,49] studied dynamic properties of the behavior of diverse types of composites, such as concrete. Tests were performed correlated with increasing material attenuation (internal friction) of the cement matrix. Promising results have been obtained as regards the vibration damping coefficient in concrete composites modified by polymers and kinescope glass [45]. In [50,51,52], concrete samples containing different proportions of sand and waste red ceramic aggregate (RWCA) were tested. Among other things, compression and bending samples were tested, crack resistance and fatigue properties were determined. The test results showed that the mechanical and fatigue properties improved or remained constant with the increase in the amount of red ceramic aggregate. Reference [53] describes the use of waste aluminum in the form of bits, powders, and dissolved substances as an alternative to alleviating the alkaline silica reaction (ARS). The authors analyzed the microstructure of the element. It has been found that both aluminum pieces and aluminum powder in various volume fractions alleviate ASR, mainly by controlling the dissolution of amorphous silica. The effectiveness of dissolved aluminum in controlling ASR decreased over time due to its easy integration with hydration products. The concrete demonstrated marked higher porosity due to gas evolution. Dynamic properties of concrete composite containing the addition of heat resistant cullet have not been covered by the testing plan.

To date, literature has not provided any studies pertaining to cement composites with heat resistant cullet as a substitute of coarse aggregate, in the context of their durability under the influence of elevated temperature and dynamic loads.

The main objective of this study was to determine possibilities of reusing waste obtained from heat resistant cullet as recycling aggregate in the production of concrete composites. This justifies the legitimacy of the undertaken issue and emphasises the cognitive and interdisciplinary nature of the study. The conducted analysis comprised selected mechanical features of the designed composite, and the compressive strength was studied.

## 2. Materials and Methods

The concrete mix was designed [54,55,56,57] based on the calculation and experimental method, with the usage of the three-equation principle. The basic components of the source mixture are as follows (Table 1): CEM I 42.5 R universal cement (Cement Ożarów S.A., Ożarów, Poland), aggregate, cat. A volatile ash (Zakład Separacji Popiołów Siekierki Sp. z o.o., Warszawa, Poland), superplastifier 18 BVC (BASF Polska Sp. z o.o., Śrem, Poland) and water. As aggregate use was made of sand with a grading of 0/2 mm (PolBot Kruszywa S.A., Warsaw, Poland) and gravel with grading of 2/16 mm (Zakład Produkcji Kruszyw Szumowo Sp. j., Szumowo, Poland). Physical, chemical, and mechanical properties of cement consistent with requirements of standard [57] have been shown in Table 2 and Table 3.

Volatile ash used in the testing came from coal burning in the Elektrownia PGNiG Termika (Warsaw, Poland). The plant in Siekierki transforms this ash into two types of products, consequently prolonging its life cycle and bringing it back in usage. The used mineral additive was certified ProAsh cat. An ash with confirmed pozzolanic properties, which meets requirements of the standard [55], and is a building product. According to relevant binding regulations it is a full-valued product. This eliminates the necessity of obtaining additional environmental decisions. It is generated by segregation of selected volatile ash during technological processing and improvement.

Table 4 presents the physicochemical parameters of fly ash type A declared by the manufacturer.

Use was made of volatile ash, since it was found that concrete with this addition (contents of 20%) offers better strength, resistance to cracking, corrosion, and high temperature [58].

The concrete was modified by a chemical admixture of 18 BVC consisting of a superplastifier based on lignosulfonates.

**Figure 1:**
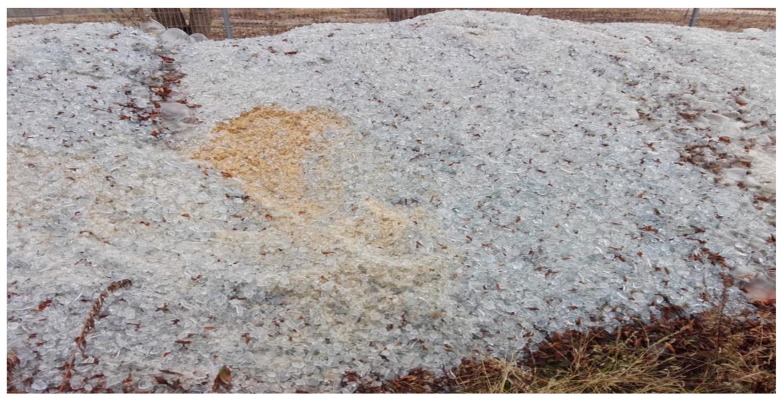
Heap with heat-resistant glass cullet formed on the premises of the Termisil S.A. Glassworks.

Test trials comprised three mixtures having different contents of glass recyclate (0, 5, and 10%). The original mixture was modified by adding heat resistant cullet to the trial batch, and consequently the amount of water and aggregate has been optimized appropriately to the volume of replaced materials, moisture contents of fine and coarse aggregate and of recyclate. When selecting recycled aggregate for needs of producing the composite material, physical parameters, and ecologic requirements were taken into consideration. Bromosilicate cullet (Figure 1) comes mainly from crushed heat resistant utensils kept in a storage area on the premises of the glass mill in Wołomin (Termisil S.A. Glassworks, Poland).

Samples for the needs of experimental testing were produced as cubes having a side length of 100 mm and stored in laboratory conditions in accordance with the standard [56]. The samples were tested to determine the selected performance properties such as compressive strength, resistance to impact strokes, high temperature, and a determination was made of the macroscopic structure of the designed composites [57,58,59,60,61,62,63,64]. The testing was conducted on a few research stands available in the Main School of Fire Service and the Lublin University of Technology.

Compressive strength testing was conducted in line with requirements of the standard [60] after 28 and 180 days of curing of concrete composite. The samples were tested at laboratory temperature of 20 °C. The measurement system comprised the controls, hydraulic press with a computer, and specialist software, which processes and records signals from the machine.

The resistance of the composite to high temperature was tested in a special furnace PK 1100/5. The samples were heated up to the temperatures of 200, 400, 600, and 800 °C, which were then maintained for 60 min until finishing of the heating process. The samples remained in the furnace until their complete cooling down.

**Figure 2:**
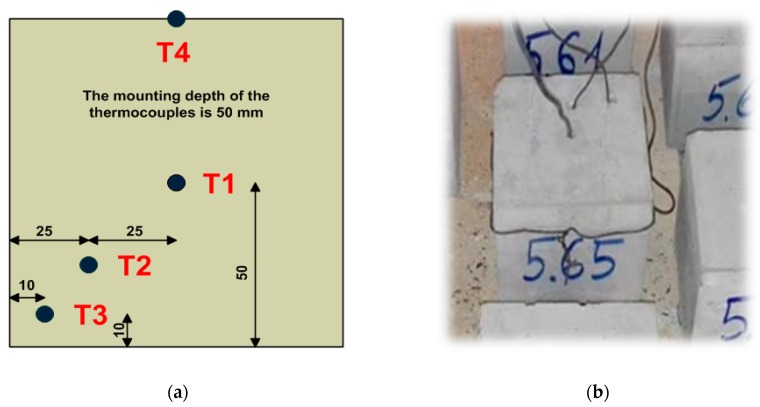
Cubic sample: (**a**) arrangement diagram for thermocouples in the sample; (**b**) view of the sample with measuring thermocouples attached.

First the samples were dried in a laboratory drying device to achieve a constant mass with temperature of 105 ± 5 °C. The signal from the thermocouples was registered by a computer furnished with dedicated software. The temperature distribution was monitored with the use of four thermoelements furnished in the specially drilled witness sample (Figure 2). The opening for the first heating element (T1) was executed in the central part of the sample, the next one (T2) 25 mm from the edge of the base and (T3) 10 mm from the base edge, at the depth of 50 mm. The last thermocouple (T4) was attached to the lateral side of the sample. The regulating thermoelement (TR) is inserted via the posterior wall and remains close to the furnace ceiling.

The testing comprised selected elements of the fire environment and the projected thermal process were programmed. Thermal fire conditions in the furnace chamber have been illustrated by the standard curve “temperature–time” [61]. Testing executed during heating of the samples conform to conditions prevailing during the fire [62].

The energy absorption testing of concrete samples was carried out at the laboratory temperature and dynamic load. Type DP FEST 1000 drop weight would hit the samples with identical energy of 150 J, from the height of 595.4 mm. The test consisted of the breaking a (100 × 100 × 100) mm cubic sample by a single hammer blow. Samples used for the testing first had to undergo thermal processing. The objective of the experiment was to measure the work, which corresponded to the energy used to break it and enabled finding ways in which the loading rate affects the fragility of material.

The scanning electron microscope (SEM) (Quanta FEG 250, Thermo Fisher Scientific, Waltham, MA, USA) was used to carry out stereologic tests of the surface of concrete composites. The testing was conducted on specially prepared microsections obtained from places where damage had been sustained. The appropriately prepared surface of the microsections was then polished with the use of sandpaper. Tests comprised studies of microstructure and phase composition of hardened concrete. In addition, an EDS (energy dispersive X-ray spectrometry) microanalysis was implemented of the chemical composition, which comprised dispersion of the X-ray energy. Chemical elements comprised by the tested material have been identified.

## 3. Results

The subject of the experiment was a population of hardened concrete composites designed by the authors. Source concrete was modified by heat resistant cullet having different cullet contents. The population was divided into three series (composite B0, B5, B10) each with one batch. Next, nine reference samples have been selected from each batch. To be able to obtain representative samples, the selection of samples for needs of the studies was random. Concrete composites were analyzed with respect to selected mechanical properties, i.e., compressive strength, resistance to elevated temperature, and dynamic impact. In addition, the structure of composites was determined using the scanning electron microscopy SEM method.

### 3.1. Average Compressive Strength

**Figure 3:**
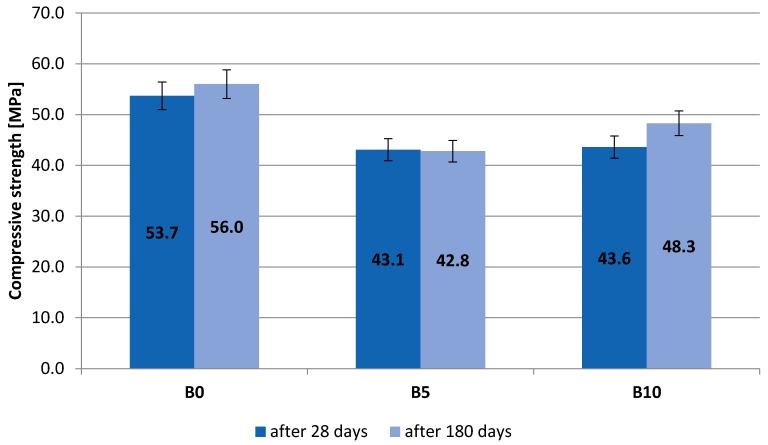
Results of compressive strength testing.

**Figure 4:**
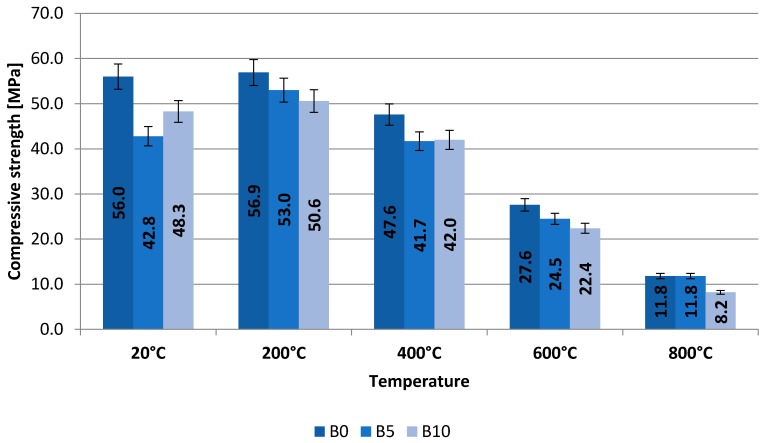
Results of testing of compressive strength obtained after 180 days.

Results of tests pertaining to compressive strength were presented in Table 5 and Table 6 and on bar charts on Figure 3 and Figure 4. The specified values are average ones obtained from three measurements. The samples were destroyed after 28 and 180 days of curing.

The rate of increase of compressive strength of the concrete mix depends on the composition of the mix. A slight increase was recorded of the average compressive strength at extended curing time of concrete composites produced with the use of recyclate. The average compressive strength B5 and B10 after 180 days amounted to 42.8 MPa and 48.3 MPa, respectively. The replacement of natural aggregate with heat resistant cullet caused a reduction of compressive strength by 23.57% and 13.75%. The target strength determined on control samples was found to be 56 MPa. In addition, the impact of recyclate on the strength parameters of the designed composite was noticed. Introducing it into the concrete mix at the level of 5% causes a decrease in the concrete strength of approx. 25% and 31% after 28 and 180 days of maturing compared to the control concrete. In the case of increasing the amount of recyclate to 10%, a smaller decrease in strength was observed, i.e., approx. 23% and 16%. The obtained test results indicate that the increase in the amount of re-rectal in the composite translates into an increase in the compressive strength of concrete.

Destructive tests were carried out on samples which had not been heated up. The conducted tests suggest that it is possible to gain a better strength of composite material by increasing the amount of recyclate in the concrete mixture.

Base samples as well as those heated with 200 °C show no changes in the internal structure. This confirms their high compressive strength. Samples of the B5 and B10 series loaded with 200 °C even showed increases in compressive strength compared to the base samples. There is some strengthening of the concrete interface. The heating of samples at a temperature of 400 °C causes a decrease in the pozzolanic activity of the binder, the formation of pores and the formation of microcracks. Increasing the soaking temperature of the samples (600 °C and 800 °C) causes the C-H-S gel to break down, increasing the size of pores and scratches, especially between the glass aggregate and the binder. You can see the crumbling of the top layer of concrete samples caused by dehydration of the C-S-H gel and the expansion of the volume of concrete caused by high temperatures. The effect of the above-mentioned factors is a change in the morphology of the concrete and, with increasing temperature, a gradual weakening of the concrete interface. Papers [65,66,67] partly confirm these explanations.

The highest strength was recorded after heating up of the samples to the temperature of 200 °C after 180 days of curing, i.e., 56.9 MPa for B0 samples, 53.0 MPa for B5 samples, 50.6 MPa for B10 samples. The compressive strength was reduced by 6.85% and 11.07% respectively. Samples heated at higher temperatures, i.e., 400, 600, and 800 °C have been found to have a bigger reduction of compressive strength as compared to samples made of control concrete. Heating up of composite in the above specified temperatures caused a decrease in strength by 12.39–11.23–0% respectively (concrete B5) and 11.76–18.84–30.51% (concrete B10). A smaller content of recyclate in the mixture has a more advantageous impact on the strength of samples heated up at fire temperatures.

### 3.2. Testing of Concrete Composite at Elevated Temperature

Pursuant to adopted assumptions, the testing was carried out in a chamber furnace in accordance with the temperature–time standard curve, conforming to the scenario of a developed fire in a closed premise, during which rescue and extinguishing actions may be carried out. Thermocouples distributed in the pilot sample allow modelling of the progress of a standardized fire. Six samples were placed at a time in the furnace and next heated up at high temperatures: 200, 400, 600, and 800 °C. The heating time depended on the height of programmed temperature.

**Figure 5:**
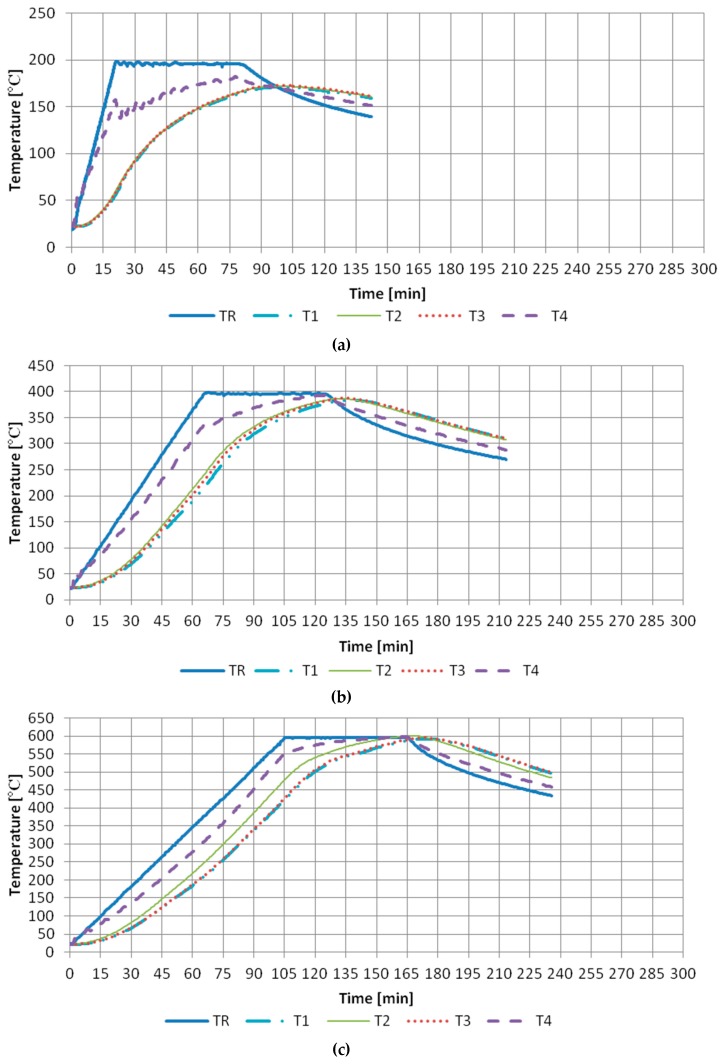

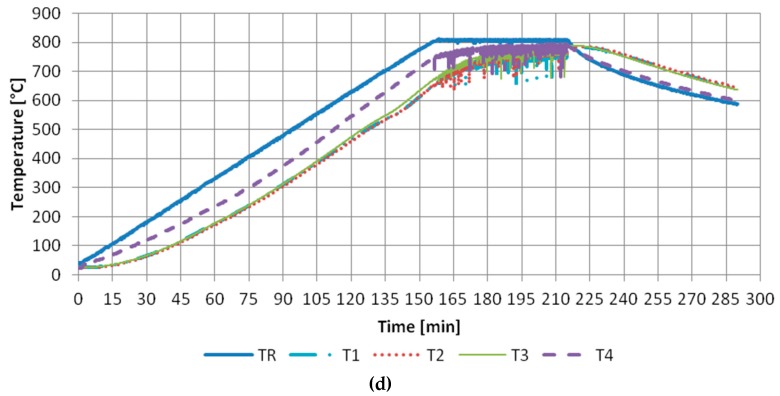
Heating curves. Temperature distribution in pilot sample for concrete composite B5: (**a**) 200 °C, (**b**) 400 °C, (**c**) 600 °C, (**d**) 800 °C.

The actual distribution of temperatures presented on Figure 5 enables the implementation of observations concerning the behavior of concrete composites in fire conditions.

The experiment has proven that the biggest temperature increase occurs over time, and in different moments of the fire the concrete composite is characterised by different strength parameters. The heated concrete goes through three phases: initial phase, temperature growth phase, and the cooling phase. In the first phase, heat from the surroundings permeates into the composite much quicker and is absorbed by the sample over its entire external surface (the air-concrete contact border). Heat that permeates inside the sample causes heating up of its subsequent layers. In the next phase, temperature grows slower. The moment when temperatures become identical proves even heating up of the sample over its entire volume. During cooling down, a temperature increase of internal layers is found to take place.

### 3.3. Impact Tests of Concrete

**Figure 6:**
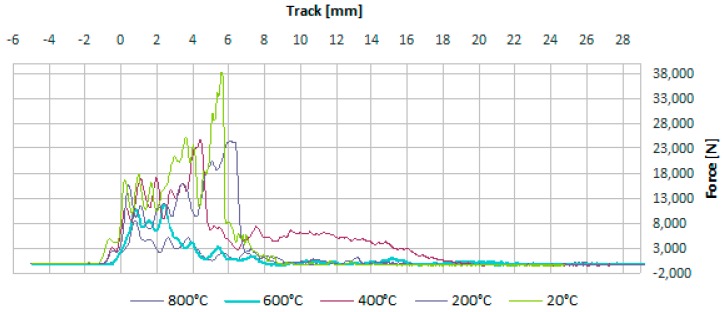
Examples of courses obtained from the implemented impact strength test: force-route.

**Figure 7:**
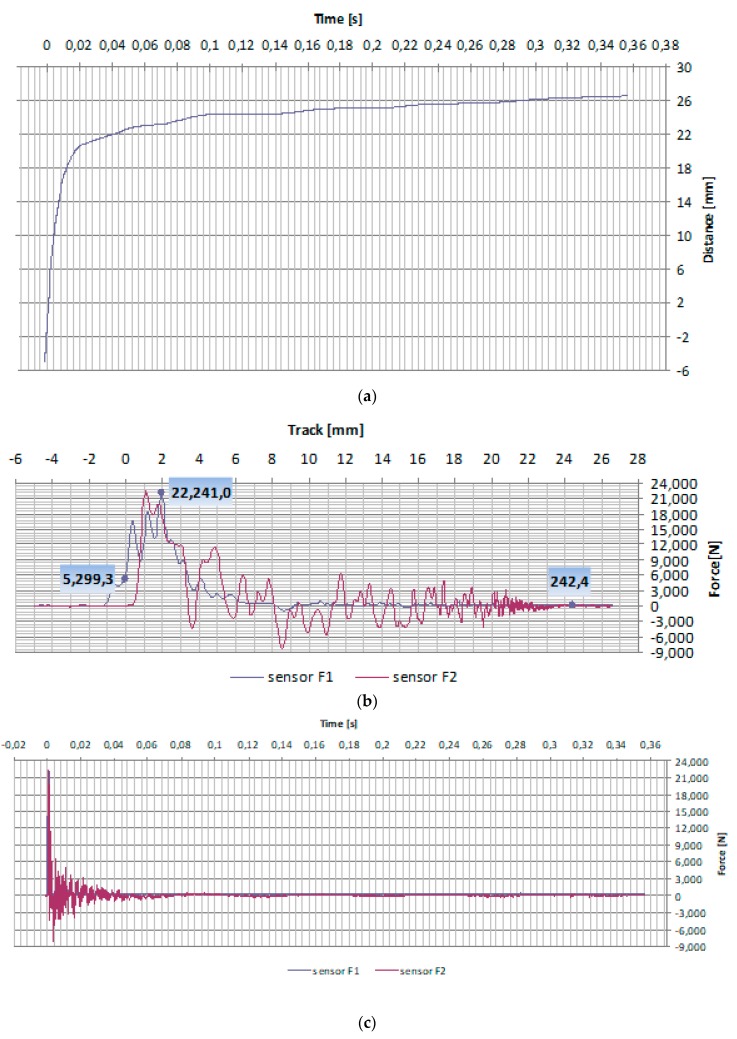
Example of results obtained during the impact strength test: (**a**) time–distance, (**b**) force–distance, (**c**) force–time.

The samples were hit by a drop hammer with energy of 150 J. The impact caused motion of particles in the concrete medium in relation to the balance situation. Results obtained from the impact test were obtained on Figure 6 and Figure 7.

As shown on Figure 7a, in the initial phase the increase of covered distance in subsequent seconds of the traffic tends to grow much quicker, and later decreases. The contained dependence of time and force is an exponential function.

As a result of stand testing (Figure 7b) the maximum value of dynamic force was obtained equalling to 22.2 kN. During dynamic impact a momentary major load increase takes place. The hammer has hit the samples with a speed of 3.30 m/s.

When analyzing the time courses of vibrations on Figure 7c a complex mechanism was recorded of energy dispersion in the concrete composite. The value of impact force of samples that had undergone stronger heating, tends to decrease, which is confirmed by the highest level of material damping of those samples. This is connected with changes to the structure of samples under the impact of temperature load, decreasing of compressive strength and rigidity, and in particular as accompanied by the appearance of cracking on the border between cullet-mortar.

### 3.4. Macroscopic Testing of the Contact Area

The analysis of the structure and gaseous phase are performed in the SEM scanning electron microscope, generally making use of zooms from 200 to 10,000×. The testing comprised in the first place sample fractures heated at the temperature of 20, 200, 400, 600, and 800 °C. The samples were taken from material left over from the process of impact stress testing.

**Figure 8:**
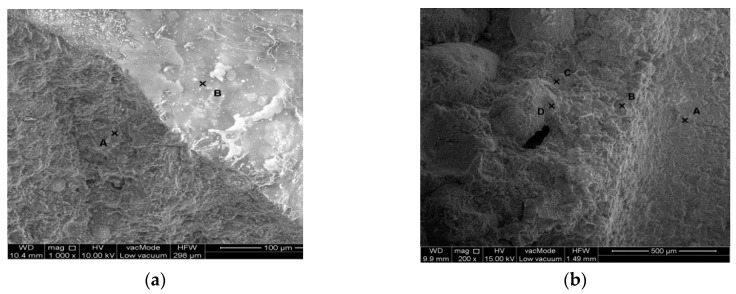
SEM photographs of microstructure of reference concrete composite (**a**) 20 °C, 0% of glass; (**b**) 800 °C, 0% of glass.

**Figure 9:**
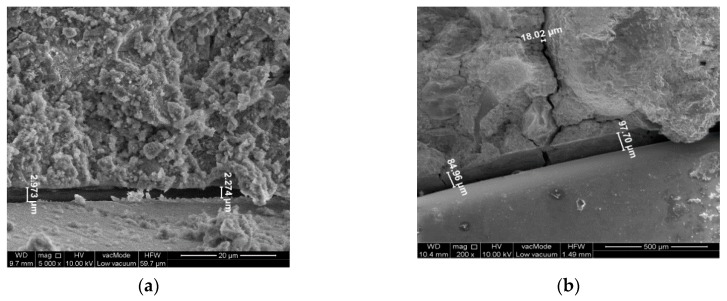
SEM photographs of microstructure of cement mortar modified by cullet: (**a**) 20 °C, 5% of glass; (**b**) 800 °C, 5% of glass.

**Figure 10:**
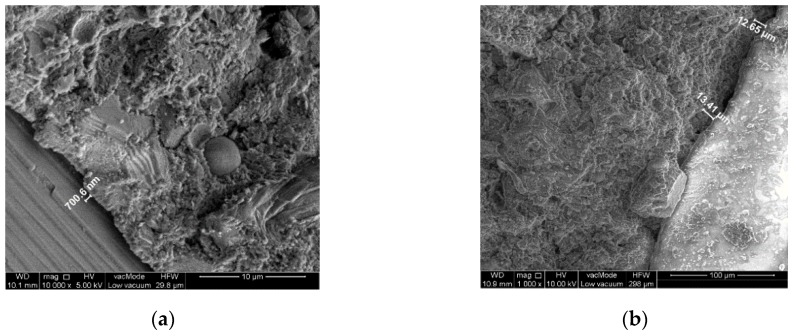
SEM photos of microstructure of cement mortar modified by cullet: (**a**) 20 °C, 10% of glass; (**b**) 800 °C, 10% of glass.

The identification of microthermic cracking and an analysis of the morphology microthermal cracking and of the morphology of concrete composites were presented on Figure 8, Figure 9 and Figure 10.

The obtained results enabled the qualitative assessment of the structure and phase composition of hardened composite. The reference concrete contains grains of hydrated calcareous silicate that is interconnected in the form of compact gel. According to Diamond’s classification [58] it conforms to morphological types III and IV of the C-S-H phase. Deformations and cracks were formed in the concrete occurring on the point of contact of the cement–aggregate matrix with residue adhesions of phase C-S-H. The cracks constitute intercluster areas of the division surface. This proves the occurrence of critical strains in the structure. Locally the material loses its strength and consistence of the matrix (Figure 8, Figure 9 and Figure 10). Cracks are recorded on the border glass aggregate—mortar (Figure 9) ranging between 2–3 µm for composite B5 heated up at 20 °C and cracks of a size of 85–100 µm for composite B5 heated at 800 °C. Composite B10 heated up at the temperature of 20 °C was found to have cracking having a width of 0.7 µm, while the temperature of 800 °C generated cracks with a magnitude of 12–13 µm.

**Figure 11:**
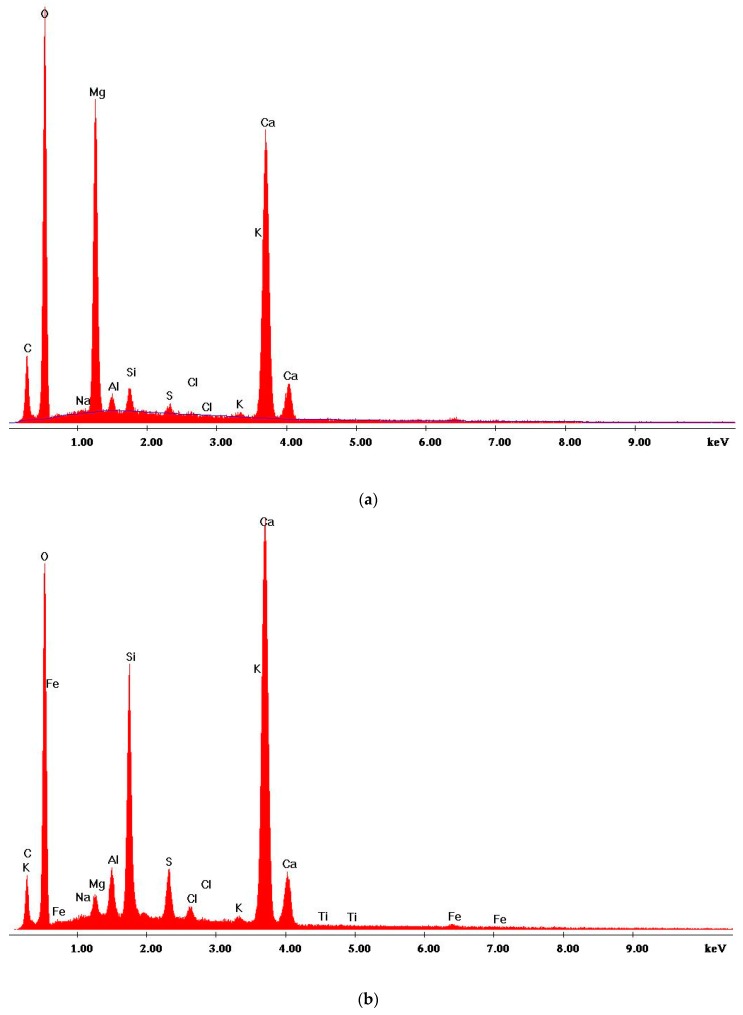
Example diffractogram of the analyzed area of modified concrete composite: (**a**) 20 °C, 5% of glass; (**b**) 800 °C, 5% of glass.

Furthermore, during structural tests of the planned composite the EDS microanalysis has been carried out (Table 7). Examples of results of the experimental X-ray spectrum on the contact area of aggregate-mortar were presented on Figure 11 as contents of particular oxides expressed as percentage.

An analysis of the above presented X-ray photograms implies that the main hydration product is the amorphous phase consisting of watered calcium silicates (gel C-S-H). Hardened concrete is characterized by high contents of silica SiO_2_ and limestone oxide CaO. In the tested samples also tested were peals which originated from crystalline intrusions of portlandite and calcite. Metakaolinite generated in the dehydration process, at a temperature of 800 °C, consists of reactive silica SiO_2_ and aluminium oxide Al_2_O_5_. When reacting with calcium hydroxide, oxides form watered calcium silicates and calcium aluminosilicates. The EDS analysis pointed to a reduction in the contents of Al_2_O_5_ by as much as 77.76% (B0, 800 °C)/12.93% (B5, 800 °C)/54.71% (B10, 800 °C) as compared to the sample of reference concrete. The SiO_2_ contents fell by 28.82%/24.15%/15.28% respectively. In an indirect way, the growth of Ca(OH)_2_ may be proven by the increased amounts of CaO equalling to 57.28%/36.84%/33.76%.

## 4. Discussion

In the tests, an attempt has been made to make a statistical assessment of results of compressive strength. Basic statistical parameters were delimited, such as mean value, median, range, standard deviation, and variability coefficient. Table 8, Table 9 and Table 10 specify the values of those parameters that characterize the homogenous nature of the designed concrete.

The conducted r-Pearson analysis suggests that increasing of the amount of recyclate and the concrete causes proportional changes (values) of the mean compressive strength. An increased amount of recyclate used to modify the amount of recyclate applied for modifying concrete in at a quantity that exceeds 10% would most probably cause an increase of its average compressive strength as compared to reference concrete.

The research of Mahesh [63], pertaining to polyethylene waste, has also provided proof of early compressive strength with a content of 5–10% of waste, their compressive strength was found to be comparable to base concrete.

**Figure 12:**
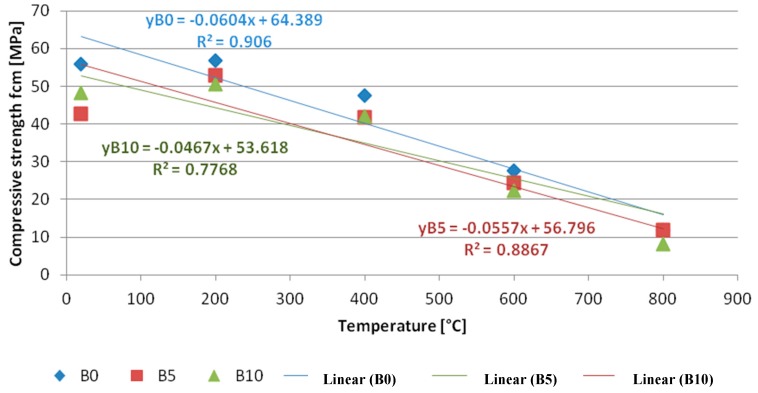
Correlation between the average compressive strength and temperature of heated concrete B0, B5, B10.

The objective of the next test was to verify the subsequent research presumption. The presumption assumed a connection of temperature at which the element with compressive strength of concrete composite is heated up. The obtained correlation was illustrated on Figure 12. The correlation was depicted as a linear function of regression (yB0, yB5, yB10) and the determination coefficient was specified (square of correlation coefficient R^2^). The variability of the average compressive strength of concrete is explained by a temperature variability of 90.60, 88.67, 77.68% respectively for the following correlation coefficients 0.95; 0.94; 0.88.

To verify the directional hypothesis, the r-Pearson analysis was performed. The regression lines were found to have a negative deviation. This proves highly negative correlation of variables, in other words a negative relation takes place between the two variables. The correlation coefficient proves the force of the interdependence. The higher the temperature, the lower the compressive strength of composites. Results of the negative correlation were found to be statistically significant, which has provided proof for the hypothesis. The test strength was determined on the basis of the adopted significance level of 0,05. The results were provided in Table 11 for bilateral testing of the hypothesis. The population number was 5.

One of the characteristic features of concrete composite is an increase of strength after heating up to the temperature of 200 °C. Also recorded was a considerable decrease in compressive strength by ca. 50% of concrete within the range of 200–600 °C. A further increase of temperature over 600 °C leads to a significant strength decrease. The composite achieves a load bearing of ca. 8–10 MPa. As has been shown by the conducted literature analysis, higher temperatures weaken the structure of composite, with surface cracking appearing. The higher the temperature affecting the material, the bigger is the decrease in the strength. Thanks to good thermal insulation, high temperature (200–800 °C) occurs only in the surface zone of the element, and does not reach the section core. The sample becomes heated up in a uniform way, which is proven by the temperature equalization moment.

The compressive strength of concrete is strongly correlated with the temperature acting on the given element. A negative relation takes place between the two variables. The result is decreasing curves of regression, determined by a linear function. The average compressive strength of concrete composite as compared to temperature change varied by 90.60% (B0), 88.67% (B5), and 77.68% (B10) respectively. The correlation coefficients amount to 0.95 (B0), 0.94 (B5), 0.88 (B10).

Heating up of the samples to a high temperature causes changes in the sample structure; the higher the temperature, the bigger the impact speed, and in such a situation weakened concrete is less resistant to impact loads. A complex mechanism of energy dispersion in the concrete becomes noticeable. Higher sample heating temperature causes a higher level of material damping.

During a comparison of structure of B0 concrete subjected to the impact of elevated temperatures to 800 °C under the SEM microscope with reference concrete at 20 °C, no scratches or delaminations have been observed in aggregate (sand) and binder. The experience of other researchers indicates that if the quantitative description of the structure is known, it is possible to define the dependence between the structure and parameters of the technological process. Measurements and an analysis of the image of concrete B5 and B10 pointed to the existence of scratching of the element. Taking into account extreme temperature loads (20 °C and 800 °C) in samples with a supplement consisting of crushed glass (5% and 10%) in the concrete mixtures, considerable differences in scratch sizes have been observed.

Differences in chemical composition of concrete subjected to the EDS analysis may arise from local differences in the material structure. The addition of volatile ash leads to a reduction in the crack opening size. The concrete compressive strength tends to drop with the increase of crack opening.

The authors are planning to undertake further research studies and carry out additional tests with the objective of learning the impact of crushed cullet on properties of concrete composites. A more extensive scope of studies would allow extending the acquired knowledge and the determination of dynamic characteristics of the planned concrete not comprised by the program under the present study and those that lack references in publications of other authors.

## 5. Conclusions

From the ecological viewpoint, the reuse of waste brings about significant economic advantages. Appropriate disposal and processing allow their re-use. Consequently, there is a need of further development of the ecological awareness of the society.

Interest in the application of cullet is growing, as it is a material that does not undergo biodegradation, and as a result this raw material may be processed many times. The studies have shown that this recyclate may constitute, in some cases, a perfect supplementing of aggregate of natural origin, as it does not require improvement and purification processing.

An analysis of the results and observations made during the executed experiment allow the following presumptions:

1. All studied concrete types achieved different classes after 180 days of curing i.e., C45/55 (B0), C30/37(B5), C35/45 (B10).
2. The average compressive strength for concrete modified by 10% of heat resistant cullet was determined for 43.6 MPa and 48.3 MPa respectively after 28 and 180 days of curing. The increase in strength over time contributed to increase the concrete class from C30/37 to C35/45.
3. The increase of temperature to 800 °C caused in all composites a change of the structure and a reduction of strength to 8–10 MPa.
4. When the sample was subjected to the temperature of 200 °C, strength has been found to grow from 1.61% to 23.83%.
5. Temperature caused damage to the concrete structure, and cracking was visible under the microscope was detected. The biggest discontinuity of the material had 90.7 µm.
6. Based on the conducted tests and analyses, it has been proven that waste of heat resistant cullet may be used again as recycled aggregate for concrete.
7. The conducting testing of the structure has pointed to minimum concentrations of selected elements. No admissible environmental standards have been exceeded, which allows the presumption that the concrete composite modified by heat resistant cullet may be considered as being a full value building material.

The concrete composite modified by waste products obtained from heat resistant cullet is found to offer satisfactory performance parameters, which has been proven during the conducted experiments.

Source materials contain little information which would describe the impact of heat resistant cullet on physical, mechanical, and chemical properties of concrete composite. Also offered information pertaining to concrete impact strength is limited. Given the absence of standard regulations concerning the undertaken research, in-depth interpretation of obtained results would require the implementation of supplementary studies. Findings presented in this article serve as a basis for further research.

## Figures and Tables

**Table 1:** Composition of control mixture in the event of weight–volume dosage in kg/m^3^.

Name of Component	Amount of Component
Cement	260
Sand	727
Gravel	1076
Volatile ash	100
Superplastifier	1.82
Water	176

**Table 2:** Physical and mechanical properties of cement CEM I 42.5 R.

Start of Binding Time (min)	Volume Stability (mm)	Proper Surface (cm^2^/g)	2-Day Compressive Strength (MPa)	28-Day Compressive Strength (MPa)
180	0.6	4189	31.1	55.4

**Table 3:** Chemical properties of cement CEM I 42.5 R as (%).

Roasting Loss	Undissolved Remnant	SO_3_	Cl^−^	Na_2_O_eq_
2.85	0.72	3.20	0.07	0.74

**Table 4:** Physicochemical composition of fly ash type A.

Component	Requirements
Appearance	grey or dark grey powder
Roasting losses	4.5%
Chlorines Cl^¯^	0.1%
Anhydride of sulfuric acid SO_3_	0.25%
Free calcium oxide CaO	0.11%
Reactive calcium oxide CaO	1.8%
Grain density	2050 kg/m^3^
Volume weakness	1 mm
Fineness	30%
Index of pozzolanic activity after 28 days	80%
Index of pozzolanic activity after 90 days	88%
Commencement of binding time as compared to reference concrete of CEM I 42.5R	285 min

**Table 5:** Average compressive strength of concrete composite fcm.

Compressive Strength	B0 ^1^	B5 ^1^	B10 ^1^
After 28 days (MPa)	53.7	43.1	43.6
After 180 days (MPa)	56.0	42.8	48.3

^1^ Concrete composite with specific contents of glass recyclate.

**Table 6:** Average compressive strength fcm (MPa) of heated concrete composite after 180 days.

Temperature	B0 ^1^	B5 ^1^	B10 ^1^
200 °C	56.9	53.0	50.6
400 °C	47.6	41.7	42.0
600 °C	27.6	24.5	22.4
800 °C	11.8	11.8	8.2

^1^ Concrete composite with specific contents of glass recyclate.

**Table 7:** Example of oxygen composition of the modified batch following the impact of thermal shock.

Oxide	Contents of Oxides as a Parentage Value (%)					
	B0 20 °C	B5 20 °C	B10 20 °C	B0 800 °C	B5 800 °C	B10 800 °C
C_2_O	8.69	24.18	13.37	11.21	7.41	17.64
Na_2_O	0.42	0.58	0.61	0.17	0.43	0.30
MgO	0.80	4.43	1.42	1.43	0.89	0.39
AL_2_O_5_	21.00	6.96	16.45	4.67	6.06	7.45
SiO_2_	32.62	23.02	15.05	23.22	17.46	12.75
SO_3_	1.49	1.28	6.87	6.09	13.52	1.97
Cl_2_O	0.39	-	0.25	0.99	-	2.16
K_2_O	1.01	0.79	0.72	0.48	0.51	0.39
CaO	31.93	37.57	42.06	50.22	51.41	56.26
TiO_2_	0.32	-	-	-	-	-
Fe_2_O_3_	1.33	1.2	3.19	1.51	2.31	0.70
Suma	100	100	100	100	100	100

**Table 8:** Basic statistical parameters. Characteristics of concrete composite after 28 days.

Symbol of Composite	B0	B5	B10
Number of data	3	3	3
Average (MPa)	53.7	43.1	43.6
Median (MPa)	53.3	43.6	43.8
Minimum value (MPa)	52.2	41.6	41.9
Maximum value (MPa)	55.5	44.0	45.1
Range (MPa)	3.3	2.4	3.2
Standard deviation (MPa)	1.37	1.05	1.31
Variability coefficient (%)	3.1	3.0	3.7

**Table 9:** Basic statistical parameters. Characteristics of concrete composite after 180 days.

Symbol of Composite	B0	B5	B10
Number of data	3	3	3
Average (MPa)	56.0	42.8	48.3
Median (MPa)	58.3	44.6	51.3
Minimum value (MPa)	50.6	37.7	41.9
Maximum value (MPa)	59.06	46.1	51.7
Range (MPa)	8.5	8.4	9.8
Standard deviation (MPa)	3.83	3.64	4.54
Variability coefficient (%)	8.4	10.4	11.5

**Table 10:** Results of the r-Pearson correlation of concrete composite after 28 days

		B0	B5	B10
**B0**	r-Pearson correlation	1.000	−0.882	0.427
	Significance (bilateral)		0.312	0.719
	N	3	3	3
**B5**	r-Pearson correlation	−0.882	1.000	0.048
	Significance (bilateral)	0.312		0.969
	N	3	3	3
**B10**	r-Pearson correlation	0.427	0.048	1.000
	Significance (bilateral)	0.719	0.969	
	N	3	3	3

**Table 11:** Results of r-Pearson correlations

		B0	B5	B10
B0	r-Pearson correlation	1.000	0.979	0.999
	Significance (bilateral)		0.004	0.002
	N	5	5	5
B5	r-Pearson correlation	0.979	1.000	0.984
	Significance (bilateral)	0.004		0.002
	N	5	5	5
B10	r-Pearson correlation	0.999	0.984	1.000
	Significance (bilateral)	0.002	0.002	
	N	5	5	5
